# Sono-Biosynthesis and Characterization of AuNPs from Danube Delta *Nymphaea alba* Root Extracts and Their Biological Properties

**DOI:** 10.3390/nano11061562

**Published:** 2021-06-14

**Authors:** Mihaela Cudalbeanu, David Peitinho, Francisco Silva, Rosa Marques, Teresa Pinheiro, Ana C. Ferreira, Fernanda Marques, António Paulo, Catarina F. Soeiro, Sílvia Andreia Sousa, Jorge Humberto Leitão, Aurel Tăbăcaru, Sorin Marius Avramescu, Rodica Mihaela Dinica, Maria Paula Cabral Campello

**Affiliations:** 1 Faculty of Sciences and Environment, Department of Chemistry Physical and Environment, “Dunărea de Jos” University of Galati, 111 Domnească Street, 800201 Galati, Romania; mihaela.cudalbeanu@ugal.ro (M.C.); aurel.tabacaru@ugal.ro (A.T.); 2 Research Center for Environmental Protection and Waste Management, University of Bucharest, 91-95 Splaiul Independentei, 050095 Bucharest, Romania; sorin_avramescu@yahoo.com; 3 Centro de Ciências e Tecnologias Nucleares, Instituto Superior Técnico, Universidade de Lisboa, Campus Tecnológico e Nuclear, Estrada Nacional 10, Km 139.7, 2695-066 Bobadela, Portugal; dpeitinho@gmail.com (D.P.); fsilva@ctn.tecnico.ulisboa.pt (F.S.); rmarques@ctn.tecnico.ulisboa.pt (R.M.); fmarujo@ctn.tecnico.ulisboa.pt (F.M.); apaulo@ctn.tecnico.ulisboa.pt (A.P.); 4 Departamento de Engenharia e Ciências Nucleares (DECN), Instituto Superior Técnico, Universidade de Lisboa, Estrada Nacional 10, 2695-066 Bobadela, Portugal; teresa.pinheiro@tecnico.ulisboa.pt; 5 Department of Bioengineering, iBB-Institute of Bioengineering and Biosciences, Instituto Superior Técnico, University of Lisbon, 1049-001 Lisbon, Portugal; catarina.soeiro@tecnico.ulisboa.pt (C.F.S.); sousasilvia@tecnico.ulisboa.pt (S.A.S.); jorgeleitao@tecnico.ulisboa.pt (J.H.L.); 6 Centro de Química Estrutural, Instituto Superior Técnico, Universidade de Lisboa, Campus Tecnológico e Nuclear, Estrada Nacional, Estrada Nacional 10, Km 139.7, 2695-066 Bobadela, Portugal; acferreira@ctn.tecnico.ulisboa.pt; 7 Department of Organic Chemistry, Biochemistry and Catalysis, Faculty of Chemistry, University of Bucharest, 90-92 Soseaua Panduri, 050663 Bucharest, Romania

**Keywords:** gold nanoparticles, *Nymphaea alba*, sonochemistry, biosynthesis, antimicrobial activity, antitumor activity, antioxidant compounds

## Abstract

Root extracts from Danube *Delta Nymphaea alba* were used to prepare gold nanoparticles (AuNPR_n_) by reducing HAuCl_4_ at different pHs (6.4–8.4) using ultrasonic irradiation: an easy, cheap, eco-friendly and green approach. Their antibacterial and anticancer activities were evaluated against *Staphylococcus aureus* and *Escherichia coli*, and A2780 ovarian cancer cells, respectively. The AuNPR_n_ were characterized concerning their phytoconstituents (polyphenols, flavonoids and condensed tannins) and gold content. All of the nanoparticles were negatively charged. AuNPR_n_ exhibited a hydrodynamic size distribution ranging from 32 nm to 280 nm, with the larger nanoparticles being obtained with an Au/root extract ratio of 0.56, pH 7 and 10 min of sonication (AuNPR_1_), whereas the smallest were obtained with an Au/root extract ratio of 0.24, pH 7.8 and 40 min of sonication (AuNPR_4_). The TEM/SEM images showed that the AuNPR_n_ had different shapes. The ATR-FTIR indicated that AuNPRn interact mainly with hydroxyl groups present in the polyphenol compounds, which also confirm their high antioxidant capacity, except for AuNPR_2_ obtained at pH 6.4. Among the AuNPR_n_, the smallest ones exhibited enhanced antimicrobial and anticancer activities.

## 1. Introduction

Nowadays, several chemotherapeutics are available for the treatment of cancer, but they all exhibit low specificity and selectivity, as well as dose-limiting toxicity. The challenge is to find more effective and less harmful therapeutic modalities for the treatment of various types of cancer. In this context, nanomaterials have the potential to revolutionize cancer therapy. In the rapidly evolving field of materials science, nanomaterials currently show leading-edge scientific advances and also fulfil many areas of human needs. In this multidisciplinary field, nanotechnology has found applications in the areas of medicine and biology owing to the unique features of nanoparticles (NPs), including their size-dependent physical and chemical properties, high chemical stability and biocompatibility [1]. Among all NPs, gold and silver nanoparticles (AuNPs and AgNPs) have attracted intensive research efforts, mostly due to their promising properties for medical applications, e.g., as cancer theranostic agents, photothermal therapy sensitizers, and antimicrobial, antiparasitic and antiviral agents [2,3,4,5,6].

The distinctive features of AuNPs for biomedical use are their unique optical properties, resulting from their characteristic surface plasmon resonance (SPR) [7]. In recent decades, various methods have been developed to synthesize AuNPs in order to modulate their SPR modes and thus improve their physico-chemical properties and their biological behavior [8]. One of the most common chemical methods to obtain AuNPs involves the use of a citrate/gold chloride system to produce AuNPs in the size range of 10–150 nm, where the citrate species acts as both reducing and stabilizing agents [8,9]. However, the size distribution, which broadens as the particle size increases, leading to highly polydispersed AuNPs, has been pointed out as one of the major drawbacks of this approach [10]. Later, the Brust method was used to obtain several AuNPs with controlled size and reduced dispersity, using NaBH_4_ as the reducing agent and by replacing the citrate with thiol, phosphine or amine ligands [10,11,12,13]. More recently, highly stable gold nanoparticles were obtained using ionic liquids, acting as stabilizing and reducing agents [14]. For all of these chemical methods, a low availability of the stabilizing agent in the solution increases the propensity to form larger particles due to Oswald ripening [15,16]. Besides these chemical methods, several physical procedures, such as sonication, laser ablation, UV irradiation and electrodeposition, have also been reported for the reduction of Au^3+^. These methods, in general, provide nanoparticles in essentially non-spherical shapes [17,18,19,20].

Although the above-mentioned methods can efficiently produce AuNPs, the generation of toxic by-products represents a major drawback, raising environmental concerns. In addition, the use of toxic chemicals and solvents can be challenging for biological applications. Therefore, new strategies to generate AuNPs without toxic chemicals and the loss of product efficiency have evolved. In this direction, biological synthetic approaches that are eco-friendly, cost-effective and suitable for scaling up have been proposed, and have emerged as promising alternatives to the conventional methods. Bacteria, fungi, viruses, algae, plants and plant extracts are among the immense range of biological resources that are available in nature, and can be applied for the successful preparation of nanoparticles based on green methods [21,22,23]. In particular, over the past few years, plants, plant extracts and other natural products have been used for the efficient and rapid green synthesis of AuNPs. The extracts can be obtained from different anatomic parts of a plant, such as leaves, roots, fruits and flowers, and represent rich sources of phytochemicals, namely polyphenols, flavonoids, alkaloids, polysaccharides, amino acids, organic acids, vitamins and proteins. All of them, particularly flavonoids, phenols, amino acids and organic acids, can work synergistically and are assumed to manifest both a reducing and stabilizing action in the preparation of AuNPs [8,24,25,26,27].

Recent studies have investigated the synthesis of AuNPs from several plants and plants extracts, predominantly leaf extracts, aiming at their application as antibacterial and anticancer agents [28,29,30,31,32,33]. Regarding bacterial infections, the occurrence of microorganisms which are becoming resistant to a high number of antimicrobial agents is a major threat to public health, and thus the application of AuNPs in the control of bacterial infections has started to be considered [17,34,35,36,37,38,39,40,41]. AuNPs synthesized using leaf extracts of *Nymphaea nouchali*, known as *Nymphaea stellate* (the blue lotus of the *Nymphaea* genus), exhibited a potent antibacterial effect against *E. coli* DH5-α [42]. The authors claimed that the effect was due to the interaction with functional proteins, resulting in protein denaturation and deactivation [42]. However, a very limited number of studies have addressed the molecular mechanisms underlying the antimicrobial activity of AuNPs. For instance, Rai et al. synthesized gold nanoparticles capped with cefaclor, which were characterized using both spectroscopic (FTIR) and microscopic (AFM) techniques [43]. These authors found that the AuNPs exerted their antimicrobial activity by the combined action of cefaclor, which inhibits the synthesis of peptidoglycan, and AuNPs that generate ‘‘holes’’ into the walls of the bacteria cells [43].

The previous reports state that, at the cellular level, NPs enter the eukaryotic cells by different pathways, such as phagocytosis, macropinocytosis, and clathrin-mediated or caveolin-mediated endocytosis. The cytotoxic effect of AuNPs might result from the physico-chemical interactions of the gold atoms, not only with the functional groups present in the intracellular proteins but also with the nitrogen bases and phosphate groups from DNA, and from decreased activities of signaling proteins [44,45]. These effects were found to be dependent on the NPs’ size and shape [46].

The anticancer activity of NPs containing plant extracts has been documented for several types of cancer cells, such as Hep2 (hepatic), HT-29 (colon), HeLa (cervical), A549 (lung) and MCF-7 (breast) cancer cells. Morphological changes, such as rounding and shrinking, were observed in Hep2 cells after treatment with AuNPs, suggesting that the cell death is mediated by apoptosis [8,47,48,49,50,51,52].

The identification of antioxidant compounds in extracts of *Nymphaea alba* from the Danube Delta biosphere, notably polyphenols and flavonoids, was previously reported [53,54]. The enhanced biological properties, such as antifungal and anticancer activities, were reported from both leaf and root extracts. These extracts showed specific activity for *Candida glabrata* and an important selective cytotoxicity against A2780 (ovarian) and MCF-7 (breast) cancer cells [54]. Hence, we studied for the first time the application of Romanian Danube Delta *Nymphaea alba* root extracts for the green synthesis of AuNPs at room temperature and the evaluation of their antioxidant, antibacterial and anticancer activity against human ovarian cancer.

## 2. Results and Discussion

There have been numerous reports during the last two decades concerning the synthesis and biological evaluation of gold nanoparticles obtained based on plant extracts. When compared to traditional chemical methods, this approach is non-toxic, environmentally friendly, and has a high cost/benefit ratio, as the extract plants are easily available. Moreover, the plant extracts act simultaneously as reducing agents for the gold and as stabilizing agents due to the presence of several different reducing agents in the plant extract. All of these advantages are certainly at the base of the emergent interest in this type of biosynthesis. The part of the plant from which the extract is extracted, such as the leaves, roots, flower, fruit juice, fruit pulp, fruit peel or seed, as well as the reaction conditions, including the pH, temperature, gold/extract ratio, light and reaction time, among others, will have an influence on the size and shape of the obtained nanoparticles, and therefore their potential biomedical usefulness [17,55,56,57]. The great challenge of the biosynthesis approach is to gather the ideal conditions that efficiently tune the shape/size of the nanoparticles with narrow and well-defined size distributions. Among the several methods that have been developed for the biosynthesis of nanoparticles, the sonication method has been used to synthesize colloidal AuNPs since the pioneering work of Baigent and Miiller in 1980 [58]. The major convenience of this method is the effective reduction in short reaction times. Besides the type of extract plant, the energy and time of the sonication are the key parameters that determine the morphology and distribution of the nanoparticles obtained through the sonication method.

Herein, we evaluated the effect of different parameters, namely the pH, the concentration of gold and root extracts, and the reaction time, on the morphology of gold nanoparticles obtained with extracts of Danube Delta *Nymphaea alba* roots as reducing and stabilizing agents, using ultrasonic energy. *Nymphaea alba* root extracts have already been evaluated (see Figure S1 and Table S1) and shown to have antifungal activity towards *Candida glabrata* and anticancer activities against A2780 (ovarian) and MCF-7 (breast) cancer cells [54]. To the best of our knowledge, this is the first study in which the ability of these extracts to produce, coat and stabilize gold nanoparticles (AuNPR_n_, n = 1–5) was evaluated. The structural properties of the different AuNPR_n_ nanoparticles were evaluated, as well as their antioxidant, antibacterial and antitumor activity, with the aim to demonstrate the potential of these extracts to produce gold nanoparticles showing a biological profile suitable to be exploited in medicinal therapy.

### 2.1. Synthesis of the AuNPR_n_ (n = 1–5)

The gold nanoparticles biosynthesized using Danube Delta *Nymphaea alba* root extracts (AuNPR_n_, n = 1–5) were obtained by sonochemistry according to the method reported by Junichi Kurawaki et al., with some modifications [55]. In the synthesis of the AuNPR_n_ (n = 1–5), different concentrations of chloroauric acid (HAuCl_4_) solution, root extracts, reaction time and pH were used. The mixtures were fixed at a constant position in an ultrasonic bath, and were subjected to ultrasound irradiation at room temperature, at a frequency of 38 kHz and a power of 100 W. The pH of the colloidal mixture was then adjusted with a 0.1 M NaOH solution, followed by mechanical stirring or sonication for additional time, at room temperature. Table 1 summarizes the different experimental conditions used to prepare the AuNPR_n_. The resulting suspensions were purified by ultra-centrifugation at 10,000 rpm for 15 min and washed three times with Milli-Q water. The obtained AuNPR_n_ were redispersed in 2 mL Milli-Q water and stored in the dark.

### 2.2. Characterization of the AuNPR_n_ (n = 1–5)

The content of polyphenols, flavonoids and condensed tannins in the root extracts and in the AuNPR_n_ were determined by a microspectrophotometric assay, whereas their Au content was determined by particle-induced X-ray emission (PIXE, High Voltage Engineering Europa B.V., Amersfoort, The Netherlands), as detailed in the experimental section. The nanoparticles were characterized by UV-visible spectroscopy (Varian, Inc., Palo Alto, CA, USA), dynamic light scattering (DLS, Malvern Instruments Ltd., Worcestershire, UK), zeta potential, attenuated total reflectance Fourier-transform infrared spectroscopy (ATR-FTIR, Madison, WI, USA), transmission electron microscopy (TEM, JEOL LTD., Tokyo, Japan), scanning electron microscopy (SEM, JEOL LTD., Tokyo, Japan) and X-ray diffraction (XRD, High Voltage Engineering Europa B.V., Amersfoort, The Netherlands). The ratios of the Au/root extract were also determined.

#### Quantification of the Polyphenol Compounds from *N. alba* Extract and AuNPR_n_

The phytoconstituent screening by microspectrophotometric methods showed the presence of secondary metabolites, polyphenols, flavonoids and condensed tannins (Table 2). The total polyphenol content was evaluated according to two different standards: gallic acid (GA) and tannic acid (TA). The total flavonoid content was assessed according to quercetin (Q) and rutin (R) standard references, and the condensed tannins content was evaluated according to a catechin (+) (C) standard. Our results highlight that, in the *N. alba* root extract, the total polyphenols, flavonoids and condensed tannins are higher than those in the AuNPR_n_ root samples due to the ability of polyphenol compounds to form a complex with the gold nanoparticles.

The gold (Au) content of the AuNPR_n_ was determined by the PIXE technique, as detailed in the experimental section. The PIXE technique is based on characteristic X-rays emitted from the sample due to relaxation of excited electron clouds by energetic proton beams delivered by particle accelerators. PIXE is a fast, multielemental and quantitative technique with high sensitivity for a wide range of elements (Z > 13), typically in the range of μg/g.

**Figure 1:**
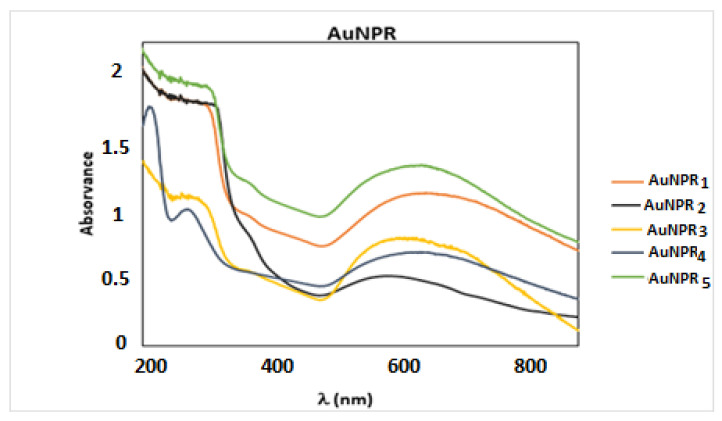
UV–Vis spectra of the AuNPR_n_ in Milli Q water.

UV–Vis spectral studies were carried out at room temperature in the wavelength range of 200–900 nm. The UV-Vis spectra of the Danube Delta *Nymphaea alba* extracts have already been published [54], and showed two absorption peaks around 200 and 300 nm, which confirm the existence of antioxidant compounds in the extracts, as flavonoids range between 240 and 400 nm. The UV-Vis spectra of AuNPR_n_ were measured in order to determine the Surface Plasmon Resonance (SPR) wavelength and infer the shape and stability of the AuNPR_n_. In fact, the SPR band is sensitive to several factors, namely the size, shape, concentration, chemical composition of the surfactant, degree of shell agglomeration, and refractive index near the nanoparticle surface. Spherical AuNPs show a characteristic SPR band around 520 nm in the visible region [7]. Figure 1 shows the UV-Vis spectra of the synthesized AuNPR_n_, and the positions of SPR bands in these spectra are given in Table 3. The SPR wavelength of all of the AuNPR_1–5_ synthesized by sonochemistry is in the range 587–628 nm. The observed red shift of the SPR band relative to that of spherical nanoparticles was ascribed to the inherent anisotropic shape of nanoparticles and/or to the formation of stable nanoaggregates/agglomerates, resulting from the interaction among the spherical nanoparticles due to Oswald ripening [14,15,56,59,60]. The AuNPR_3_ and AuNPR_4_ samples also displayed two absorption bands at <350 nm corresponding to the polyphenolic molecules of the root extract [61], which suggests that the polyphenols present in the root extract act as an effective stabilizer in these two samples, which were prepared with a higher concentration of root extracts. For AuNPR_2_, the same was expected, although these bands are not apparent. We hypothesized that in the case of AuNPR_2_, the reaction conditions—namely the reaction pH (6.4)—did not allow the conjugation of polyphenols or flavonoids to the nanoparticles.

In order to evaluate the stability of the nanoparticles in time, UV-Vis spectra were recorded regularly for three months (once a week). Prior to the acquisition of the UV spectra, the nanoparticles were ultrasonicated for 2 min at room temperature. It should be noticed that the UV–Vis spectra of all of the AuNPR_n_, recorded in Milli Q water, did not show significant changes either in intensity or in the SPR absorbance peak over the three months. Thus, the observed red-shift on the absorption maxima of the SPR bands reflects the tendency of the AuNPR_n_ to form larger nanoparticles and/or agglomerates in a solution, because the formation of aggregates would lead to obvious changes in the UV-Vis spectra [62,63].

The DLS analyses of the AuNPR_n_ showed different hydrodynamic sizes of between 32 nm and 280 nm. As can be seen Table 3, the hydrodynamic size is strongly dependent on the ratio of the Au/root extract, with the higher ratios leading to higher hydrodynamic size values. The reaction pH and the sonication time are also conditioning factors for the particle size, although they are not so obvious. It seems that short sonication times and a pH lower than 7.0 promote the increase of the hydrodynamic size. However, with respect to AuNPR_5_, the Au/root extract ratio seems to be the prevalent factor, surpassing the influence of these parameters. The average polydispersity index (PDI) was 0.254, which is consistent with a moderate monodispersed distribution, in good agreement with the shape and size heterogeneity of the AuNPR_n_ dispersions. AuNPR_4_ presents the lowest hydrodynamic size but also the highest PDI, which reflects the involvement of biological material from the root extracts surrounding the nanoparticles (see Figure S2 and Table S2).

Zeta potential indicates the electrical charge on the surface of synthesized NPs, and is also a key factor used to predict the stability of AuNPs in terms of monodispersity (or agglomeration) [64,65,66]. Values above ± 60 mV are indicative of a high stability, whereas values between 0 mV and ± 5 mV are indicative of strong instability [64]. As shown in Table 3, the zeta potential of all of the synthesized AuNPR_n_ was negative, comprising the range −62 ± −11 mV; −46 ± −7 mV, corroborating the stability of the nanoparticles [64]. Regarding the other gold nanoparticles formed with plant extracts, the synthesized AuNPR_n_ presents lower ζ values [65]. Several reports in the literature show the dependence of the zeta potential on the physico-chemical properties of the particle surface and on the nature of the solution. For instance, small changes in the ionic strength and pH can lead to large effects on the zeta potential [64,65,66]. In this study, the zeta potential decreased with the increase of the reaction pH; it can be seen that the AuNPR_2_ stabilized at pH 6.4 presented the highest ζ, while the AuNPR_3_ stabilized at pH 8.4 had the lowest zeta potential value (Table 3). Similar results were observed in the green-synthesis of other AuNPs [65,66,67].

**Figure 2:**
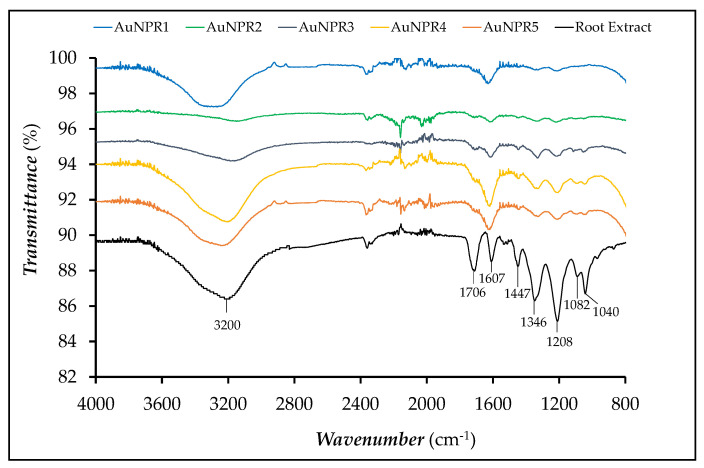
ATR-FTIR spectra of the root extract and all of the AuNPRn samples.

The ATR-FTIR spectrum of the pure root extract and AuNPR_n_ samples are shown in Figure 2. The aqueous root extract presented IR absorption regions characteristic of polyphenol compounds. Bands at 3200 cm^−1^, related to hydroxyl groups, could be observed, whereas the bands related to the stretching vibration of the OH groups and to the OH wagging in phenolic compounds were not identified. The bands at 1706 and 1607 cm^−1^ were assigned to the stretching vibrations of the C=O and C=C groups, which are associated to carboxylic acid groups and aromatic rings, respectively, present in flavonoids and other polyphenol compounds. The band at 1447 cm^−1^ could be assigned to C–O–H in the plane bending of hydroxyl groups in flavonoids. The band at 1346 cm^−1^ was attributed to the C–O stretching of the acidic groups or to the bending vibrations of –CH_3_ or –CH_2_ groups in carboxylic acids [68]. The band located at 1208 cm^−1^ was ascribed to the vibration of the C–O group in polyols, such as hydroxyflavonoids, whereas the bands located at 1082 and 1042 cm^−1^ were ascribed to secondary alcohols and/or to the stretching vibrations of the –C–O–C group in esters [69,70,71].

Almost all of the bands were observed for all of the AuNPR_n_ samples in the same range of wavenumbers [72]. However, it could be observed that the band at 1706 cm^−1^ underwent a significant decrease of its intensity. From the data of the ATR-FTIR, it can be inferred that the acidic groups of the polyphenol compounds remained chemically attached to the surface of AuNPR_n_ through the acidic functionality, which may be due to the OH groups present in the polyphenol compounds, which are involved in the reduction of Au^3+^ ions to Au^0^ [71]. The analysis of the FTIR spectra also confirmed the very weak concentration of flavonoids and polyphenols in the AuNPR_2_ sample.

**Figure 3:**
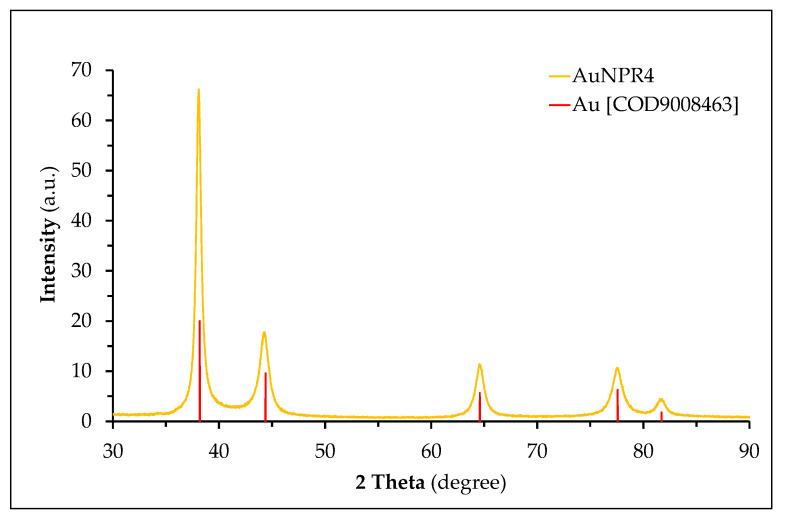
PXRD patterns of the AuNPR_4_ sample.

A PXRD analysis was conducted in order to assess the nature of the crystallinity of all of the AuNPR_n_ samples (Figure S3). The PXRD pattern of AuNPR4 (Figure 3) revealed the presence of five diffraction peaks that were indexed to the (111), (200), (220), (311) and (222) lattice planes specific to the face-centered cubic (FCC) structure of metallic gold, in agreement with the Crystallography Open Database (COD 9008463). The absence of other crystalline phases confirms the pure crystalline nature of all of the AuNPR_n_. The crystallite size of the gold core in the nanoparticles was determined by means of DIFFRAC.EVA software (Bruker AXS version 5.1.0.5, Madison, WI, USA) through Scherrer’s equation, using the diffraction plan (111) (Table 4).

Although the experimental conditions used in the biosynthesis of the nanoparticles did not translate into marked differences in the pattern of the XRD diffractograms, slight shifts in the diffraction peak positions were observed, reflecting the differences found in the dimensions of the gold core. They presented an average core size lower than 20 nm (AuNPR_4_ < AuNPR_3_ ≈ AuNPR_2_ < AuNPR_5_ ≈ AuNPR_1_), which is in good agreement with results from other nanoparticles synthesized with plant extracts [73,74,75,76,77,78,79,80], although it seems to be independent of the root extract concentration. The observed core size of the AuNPR_n_ is much smaller than the respective measured hydrodynamic size, which is due to the fact that the hydrodynamic diameter also reproduces the presence of the coating of biomolecules from the root extracts around the nanoparticle core, whereas the crystallite diameter is restricted to its core diameter. 

**Figure 4:**
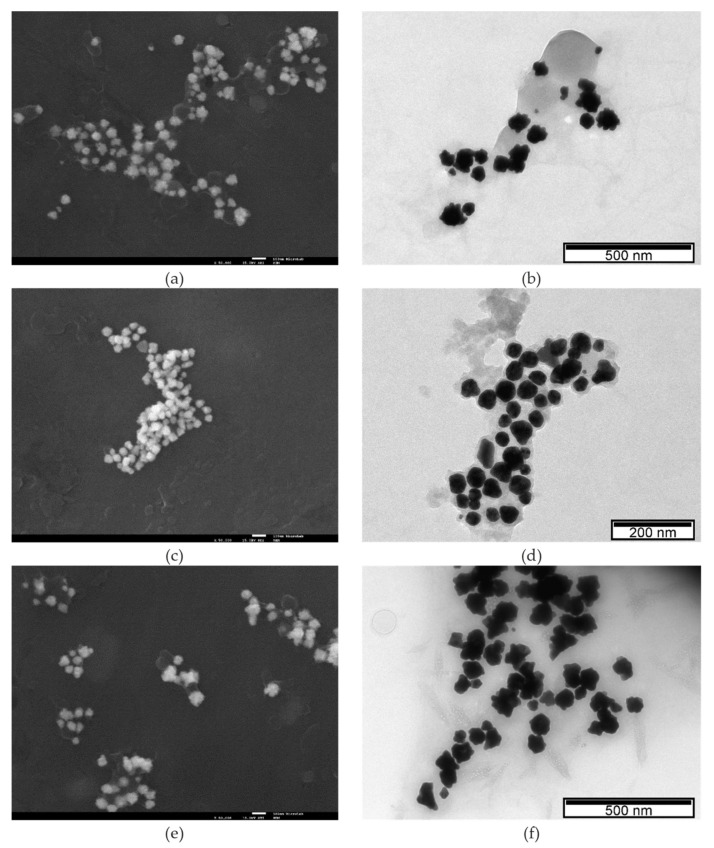
SEM and TEM images of AuNPR_1_ (**a**,**b**), AuNPR_4_ (**c**,**d**) and AuNPR_5_ (**e**,**f**). SEM images ×50,000 magnification.

SEM and TEM analyses were performed for the morphological characterization of the biosynthesized nanoparticles in terms of shape and size distribution. Figure 4 shows representative SEM and TEM images of AuNPR_n_. In Figure S4, the TEM images of the remaining AuNPR_n_ samples are presented. The SEM (Figure 4a,c,e) and TEM (Figure 4b,d,f) images show that the formed AuNPR_n_ have different shapes, coexisting in quasi-spherical, trianglular and star forms, among other irregular shapes, as well as a wide size distribution, which supports the UV-Vis spectra pattern, DLS and TEM results. Although the nanoparticles are well dispersed, they are surrounded by a thin layer of root extracts. The coexistence in the colloidal solution of nanoparticles of a great diversity of morphologies, in which the nanoparticles are surrounded by a bioorganic matrix, is a common characteristic and is attributed to a protective and stabilizing function preventing the aggregation of nanoparticles [77,78,79,80,81,82,83]. The average particle size obtained by the TEM analysis was higher than that obtained from the analysis of the diffraction patterns using Scherrer’s equation (Table 2). In fact, it was not expected that these values would coincide, as PXRD measures the crystal size (crystallite size), while electron microscopy gives information about the particle size. Thus, the larger the size and shape distribution of the nanoparticles in the colloidal solution, the greater the discrepancy of the values found by these two techniques [84,85,86]. Considering the size measurements of AuNPs from *Nymphaea alba* root extracts from the Danube Delta under different reaction conditions, an order can be established depending on the technique used: DLS > TEM > XRD. This fact is also not unusual for nanoparticles synthesized with plant extracts [87,88,89,90]. The observed crystallite size and particle core size of AuNPR_n_ were much smaller than the respective measured hydrodynamic size, which is due to the fact that the hydrodynamic diameter also reproduces the presence of the coating of biomolecules from the root extracts around the core of the nanoparticles.

### 2.3. Biological Studies

#### 2.3.1. Antioxidant Activity

A DPPH free radical assay was employed in order to evaluate the antioxidant activity of the *N. alba* root extract and AuNPR_n_. As can be observed in Table 5, the AuNPR_n_ showed a more pronounced antioxidant activity than the *N. alba* root extract, with the exception of AuNPR_2_. This difference certainly reflects the low content of root extract of the AuNPR_2_.

#### 2.3.2. Antibacterial Activity of Gold Nanoparticles (AuNPR_n_)

The antimicrobial properties of all of the AuNPR_n_ samples and the gold salt HAuCl_4_ were assessed towards the Gram-positive bacteria *S. aureus* Newman and the Gram-negative *E. coli* ATCC25922 by determining the minimum inhibitory concentration (MIC) values using the microdilution method (Table 6).

For the evaluation of the capacity of these nanoparticles as antibacterial and anticancer agents, it was important to normalize the amount of gold in each preparation, so that their biological activities can be compared. The quantification of the gold was achieved by particle-induced X-ray emission (PIXE).

The Au (III) salt precursor HAuCl_4_ exhibited a high antimicrobial activity against *S. aureus* Newman and *E. coli* ATCC25922, with estimated MIC values of 50 μg Au/mL and 6.25 μg Au/mL, respectively. The gold nanoparticle AuNPR_4_ had the highest antimicrobial activity against *S. aureus* Newman, with an estimated MIC value of 100 μg Au/mL. The AuNPR_4_ also exhibited antimicrobial activity against *E. coli* ATCC25922, with an estimated MIC value of 200 μg Au/mL. These results agree with other reported results, demonstrating an inverse relationship between the antibacterial activity and the nanoparticle size. In fact, AuNPR_4_ exhibited the lowest hydrodynamic size [85].

The gold nanoparticles AuNPR_1_ and AuNPR_3_ also exhibited antimicrobial activity against *S. aureus* Newman, with AuNPR_3_ also being active against *E. coli* ATCC25922. For the gold nanoparticle concentrations tested, the AuNPR_2_ and AuNPR_5_ nanoparticles exhibited no antimicrobial activity, with AuNPR_1_ also being inactive against *E. coli* ATCC25922. 

The lowest antibacterial activity was found for AuNPR_2_. In fact, these NPs were synthesized at a lower pH (6.4) and presented a high hydrodynamic size. In addition, the total polyphenol and flavonoid content were very low or negligible (Table 2) due, most probably, to the lower pH hampering the linkage of the root extract molecules to the AuNPR_n_ surface.

#### 2.3.3. Anticancer Activity

**Figure 5:**
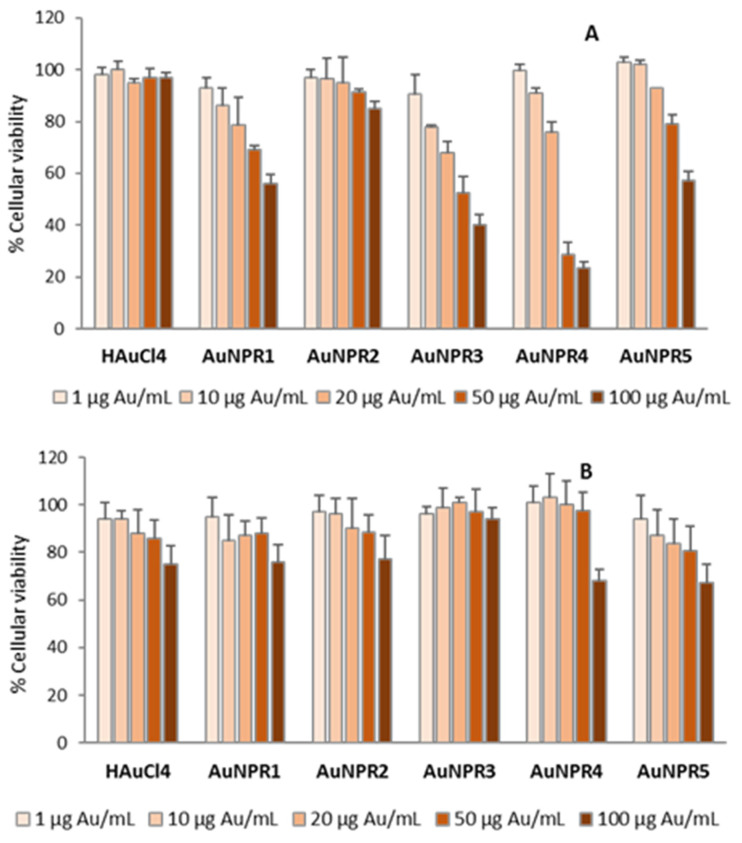
The viability of A2780 cells (**A**) and V79 fibroblasts (**B**) after treatment with serial concentrations of AuNPR_n_ for 48 h. The results are the mean ± SD of two independent experiments performed with six replicates per condition.

The cytotoxic effect of the biosynthesized AuNPRn against a cancer cell model was evaluated in order to obtain information regarding the prospective value of these nanoplatforms as chemotherapeutic agents. The cytotoxicity of AuNPs synthesized with plant extracts is dependent on the particle’s physicochemical properties, namely its size, shape, surface charge and phytochemical constituents. All of them play key roles in the cellular uptake and the degree of cytotoxicity. Several studies have shown that smaller AuNPs are the more cytotoxic. Nevertheless, the viability assay used and the type of cell line are also determinant criteria in the cytotoxicity evaluation [91,92]. The MTT assay, a metabolic assay considered the “gold standard” for cytotoxicity, was selected for the anticancer evaluation. The results obtained by the MTT assay in the A2780 cells treated for 48 h with serial dilutions of the AuNPR_n_ (1 µg Au/mL to 100 µg Au/mL) and the precursor tetrachloric acid are presented in Figure 5. The results exhibited a concentration-dependent decrease in the viability of the A2780 cells with the increase of the AuNPR_n_ concentration. The IC_50_ values, calculated from dose–response curves (Figure 5A and Figure S5a), amounted to 108.7 ± 15 µg Au/mL, >>100 µg Au/mL, 51.9 ± 7.7 µg Au/mL, 33.5 ± 6.3 µg Au/mL and 136.1 ± 15 µg Au/mL for AuNPR_1_, AuNPR_2_, AuNPR_3_, AuNPR_4_ and AuNPR_5_, respectively. The precursor gold salt was also tested, and was without cytotoxic effect.

AuNPR_3_ and AuNPR_4_ presented the lowest IC_50_ values of the series. These NPs were able to inhibit the viability of A2780 cells by 50% at 33.5 ± 6.3 µg Au/mL and 51.9 ± 7.7 µg Au/mL concentrations. These nanoparticles have a smaller hydrodynamic size and core size relative to the other NPs evaluated in this work, which might justify their enhanced anticancer activity. The cytotoxic selectivity of the AuNPR_n_ against cancer cells versus normal cells was evaluated in normal V79 fibroblasts. As can be observed from Figure 5B, a dose-dependent effect was observed even for the gold salt. However, and in particular for AuNPR_3_ and AuNPR_4_, the cytotoxic effect was less extensive, with IC_50_ values far superior to 100 µg Au/mL (Figure S5b).

To the best of our knowledge, there are only a few reports on the evaluation of the anticancer activity of gold nanoparticles stabilized with root extracts when compared to leaf extracts; therefore, it is not straightforward to compare our results directly with related nanoparticles [17]. However, it is worthwhile to mention that spherical gold nanocomposites stabilized with fungal asparaginase, ranging from 20 to 50 nm in size, showed only a minor cytotoxic effect against the A2780 ovarian cancer cell line. The cytotoxicity (%) increased from 11.92 to 18.51% with the increase in the gold nanobiocomposite concentration from 25 to 1000 μg/mL [93]. Arvizo et al. reported that 20 nm gold nanoparticles, stabilized with sodium citrate, presented a great efficiency in the inhibition of the proliferation of A2780 cells after 72 h incubation. The 5 nm AuNPs congener showed only a modest inhibition, while AuNPs of 50 and 100 nm had no cytotoxic effect. This trend is consistent with the results that we have obtained for the AuNPR_n_ stabilized by the Danube Delta *Nymphaea alba* root extracts [94].

The properties of AuNPs, such as size, charge and surface chemistry, have been shown to affect the uptake and cytotoxicity of AuNPs into cells, as well as their intracellular fate. Particle size is one of the most important factors in determining the interactions of AuNPs with biological systems, influencing their uptake and cytotoxicity. Generally, data on the size-dependent effects of AuNPs are inconclusive, which might be due to the variations of different factors, such as the shape, surface coating and agglomeration of AuNPs, as well as the cell type, incubation time and other factors such as the experimental conditions. Our results indicate that the physico-chemical properties of the AuNPs, such as the size, shape and cell type (cancer cells vs. normal cells) are critical determinants of the cytotoxicity after 48 h incubation. These results seemed to be somewhat in agreement with the other reports and the experimental conditions used to evaluate the cytotoxic activity [95,96].

AuNPs with different sizes may enter cancer cells or normal cells in different ways, such as transmembrane diffusion, protein channels or receptor-mediated endocytosis, as multiple protein receptors are overexpressed on the surface of cancer cells. The cytotoxicity could be related to the intracellular localization of the AuNPs. AuNPs with size <100 nm are thought to enter via receptor-mediated endocytosis. Nevertheless, several experimental factors can mediate the uptake and cytotoxicity. To cite a few parameters of the cellular uptake and cytotoxicity: (i) they are dependent upon the amount of gold, as the uptake is higher for high AuNPs concentrations; (ii) both cancer cells and normal cells have similar uptakes only at lower AuNPs concentrations; (iii) the uptake and cytotoxicity of AuNPs with smaller size (e.g., 5 nm) is similar for both cancer and normal cells, while for sizes higher than 50 nm the cellular uptake and the cytotoxicity is higher for cancer cells than normal cells; (iv) the uptake depends on the incubation time, in that, for shorter incubations, the uptake is similar both for cancer and normal cells, but for longer incubations the uptake into cancer cells is higher.

Based on our study, the AuNPs’ cytotoxicity is size- and cell type-dependent. However, the mechanism underlying these effects needs to be investigated in future works.

## 3. Materials and Methods

### 3.1. General

All of the chemicals and solvents (reagent grade) were purchased from Sigma-Aldrich (St. Louis, MO, USA), and were used without further purification, unless stated otherwise. The solvents for the high-performance liquid chromatography (HPLC) were HPLC-grade. Milli-Q (DI) water (ρ < 18 MΩ) was used for both the preparation of the aqueous solutions and the rinsing of the synthesized AuNPs. The roots of the Danube Delta *Nymphaea alba* species were obtained from the Biosphere Reserve of Romania. The root extracts were prepared at the Faculty of Sciences and Environment, Department of Chemistry, Physics and Environment, “Dunărea de Jos” University of Galati [53]. The A2780 ovarian cancer cells line was obtained from ATCC, Manassas, VA, USA.

### 3.2. Biosynthesis of Gold Nanoparticles by Danube Delta Nymphaea alba Roots Extracts (AuNPR_n_) Using a Sonochemistry Methodology

#### 3.2.1. Plant Material and Extract Preparation

The *N. alba* samples were authenticated and placed in the Botanical Garden of Galati, Romania. The *N. alba* root was collected in June 2017 from the Somova-Parches Lagoon Complex, Danube Delta Biosphere Reserve, Romania [53,54]. Distilled water and ultrapure water were used to perform the initial washing of the root sample. Then, the sample was dried at room temperature to a constant weight and ground to obtain a homogenous sample (granulometry lower than 2 mm).

The *N. alba* root extraction and fractionation was performed by mechanical agitation for 24 h. First, 100 g of the root sample was extracted with 2000 mL 99% cyclohexane, 0.6% ethyl acetate and 0.4% butanol. The extract was subjected to a first filtration using cotton, and then to a second filtration using a quantitative filter paper. After that, the doubly filtered extract was concentrated under reduced pressure in a rotary evaporator for solvent removal. Finally, the root residue was resumed with ethanol, followed by filtration and evaporation as in the previous procedure. The *N. alba* root extracts were placed in brown glass tubes and stored in the refrigerator at 4 °C.

#### 3.2.2. Preparation of the Root Extracts Used for the NP Synthesis

In total, 60 mL Milli-Q water was used to disperse 1 g powdered root extract under stirring for 1 h at 30 °C. The obtained dispersion was cooled down to room temperature, subjected to double filtration with Whatman No. 1 filter paper, and used immediately.

#### 3.2.3. General Procedure for the Synthesis of the Gold Nanoparticles

The gold nanoparticles were synthesized according to the method reported by Junichi Kurawaki et al., with some modifications [55]. In the synthesis of the AuNPR_n_ (n = 1–5), different concentrations of chloroauric acid (HAuCl_4_) solution, root extracts, reaction times and pH were used. The mixtures were fixed in an ultrasonic bath (240 × 137 × 150 mm) (FisherbrandTM S-Series FB15051, operating frequency 38 kHz, power 100 W), and were subjected to ultrasound irradiation at room temperature. The pH of the colloidal mixture was then adjusted with a 0.1 M NaOH solution and sonicated for additional time at room temperature. The resulting suspensions were finally subjected to centrifugation at 10,000 rpm for 15 min, and were washed three times with Milli-Q water. The obtained AuNPR_n_ was re-dispersed in 2 mL Milli-Q water and stored in the dark. 

Regarding AuNPR_1_, to 15 mL aqueous solution containing 5 mL root extract solution, 225 µL 0.1 M HAuCl_4_ was added ([Au] = 1.5 mM). After subjecting the mixture to ultrasound irradiation, the color of the resulting solution changed almost immediately from brown-yellow to grey-blue. After 10 min, 10 µL 0.1 M NaOH solution (pH = 7) was added and the final solution was mechanically stirred vigorously for further 4 h.

Regarding AuNPR_2_, to 20 mL aqueous solution containing 9 mL root extract solution, 300 µL 0.1 M HAuCl_4_ ([Au] = 1.5 mM) was added. After subjecting the mixture to ultrasound irradiation, the color of the solution changed almost immediately from brown-yellow to grey-purple. After 10 min, the pH was adjusted to 6.4 by adding 2 µL 0.1 M NaOH solution.

Regarding AuNPR_3_, to 20 mL aqueous solution containing 9 mL root extract solution, 300 µL 0.1 M HAuCl_4_ ([Au] = 1.5 mM) was added. After subjecting the mixture to ultrasound irradiation, the color of the solution changed almost immediately from brown-yellow to blue-purple. After 10 min, 20 µL 0.1 M NaOH solution (pH = 8.4) was added, and the final solution was left under ultrasound irradiation for further 30 min. 

Regarding AuNPR_4_, to 18 mL aqueous solution containing 9 mL root extract solution, 360 µL 0.1 M HAuCl_4_ ([Au] = 2 mM) was added. After subjecting the mixture to ultrasound irradiation, the color of the solution changed almost immediately from brown-yellow to blue-purple. After 10 min, 13 µL 0.1 M NaOH solution (pH = 7.8) was added, and the final solution was left under ultrasound irradiation for a further 30 min. 

Regarding AuNPR_5_, to 15 mL aqueous solution containing 5 mL root extract solution, 18 µL 2.5 M HAuCl_4_ ([Au] = 3 mM) was added. After subjecting the mixture to ultrasound irradiation, the color of the solution changed almost immediately from brown-yellow to dark purple. After 10 min, 19 µL 0.1 M NaOH solution (pH = 7.8) was added, and the final solution was left under ultrasound irradiation for further 30 min.

### 3.3. Characterization of Gold Nanoparticles (AuNPR_n_ (n =1–5))

#### 3.3.1. Quantification of the Polyphenol Compounds, Total Flavonoids and Total Condensed Tannins from *N. alba* Extract and AuNPR_n_

• HPLC-DAD chromatography analysis of the *N. alba* extract

High-Performance Liquid Chromatography with a diode-array detector (HPLC-DAD, RIGOL TECHNOLOGIES, INC Beijing, China) was used for the identification and quantification of the total polyphenol content in the *N. alba* ethanolic root extract. In total, 10 μL extract was injected into the HPLC system and the compounds were chromatographed over a Kinetex EVO C18 column (150 × 4.6 mm, particle size = 5 µm) at 30 °C. The following combination of solvent mixtures was used: H_2_O + 0.1% TFA or solvent A, and ACN + 0.1% TFA or solvent B. The flow rate was 1 mL/min, the gradient elution ranged from 2% to 100% B, and the time was set to 60 min. The analyses regarding the identification and quantification of the total polyphenol content in the *N. alba* ethanolic root extract were performed by comparison with standard spectra at each retention time. The stock solutions containing the reference compounds were prepared so that their concentration was 1000 μg/mL. For the calibration curves, concentrations between 10 and 400 μg/mL were used. The total polyphenols were analyzed at 300 nm detection [97,98,99].

• Total polyphenol content of *N. alba* extract and AuNPR_n_

The total polyphenol content of the *N. alba* extract and AuNPR_n_ were quantified by the Folin–Ciocalteu method, as follows: to 10 µL of the samples (*N. alba* root extract and AuNPR_n_), 25 µL of 1 M Folin-Ciocalteu reagent was added. Then, the samples were left in the dark for 5 min. After this time, 25 µL 20% Na_2_CO_3_ solution and ultrapure water were added until the final volume was 200 µL. The replacement of the Folin-Ciocalteu reagent with ultrapure water allowed us to obtain the blank samples. After 30 min of standing at room temperature, the samples were placed in a Tecan Pro 200 multiwell plate reader (Tecan, Männedorf, Switzerland) and their absorbance was recorded at 760 nm. As the standard references, gallic acid (0.97–500 µg/mL) and tannic acid (0.97–500 µg/mL) were used, and the results were expressed as equivalents of gallic acid (mg GAEq/g) and tannic acid (mg TAEq/g) per 1 g of sample [97,98,99].

• Total flavonoid content of the *N. alba* extract and AuNPR_n_

The total flavonoid content of the *N. alba* extract and AuNPR_n_ were quantified by the colorimetric assay involving aluminum chloride, as follows: to 100 µL of the samples (*N. alba* root extract and AuNPR_n_), 100 µL 2% AlCl_3_ solution was added. After 15 min of standing at room temperature, the samples were placed in a Tecan Pro 200 multiwell plate reader (Tecan, Männedorf, Switzerland) and their absorbance was recorded at 415 nm. AlCl_3_ and ultrapure water were used to prepare the blank samples. As the standard references, quercetin (0.45–250 µg/mL) and rutin (0.45–250 µg/mL) were used, and the results were expressed as equivalents of quercetin (mg QEq/g) and rutin (mg REq/g) per 1 g of the sample [97,98,99].

• Total condensed tannin content of the *N. alba* extract and AuNPR_n_

The total condensed tannin content was measured according to a previous study [47]. Briefly, to 10 µL of the samples (*N. alba* root extract and AuNPR_n_), 150 µL 4% vanillin-containing methanolic solution and 75 µL concentrated HCl (36%) were added. For the blanks, the samples were replaced with 10 µL ultrapure water. After 15 min of standing at room temperature, the absorbance values were recorded at 500 nm using the Tecan Pro 200 multiwell plate reader (Tecan, Männedorf, Switzerland). As the standard reference, catechin (+) (10–100 µg/mL) was used, and the results were expressed as equivalents of catechin per 1 g of the sample (mg CEq/g).

#### 3.3.2. Determination of the Gold Content in AuNPR_n_

The gold (Au) content of the obtained nanoparticles was quantified by the particle-induced X-ray emission (PIXE) technique at 2.5 MV on the IST of the Van de Graaff accelerator. The analyses were carried out with a 2.0 MeV proton beam. The PIXE technique is based on the characteristic X-rays which are emitted from the sample due to the relaxation of excited electron clouds by energetic proton beams delivered by particle accelerators. PIXE is a fast, multielemental and quantitative technique with high sensitivity for a wide range of elements (Z > 13), typically in the range of μg/g. The detailed methodology involving the PIXE set-up, quantitative analysis and sample preparation was described elsewhere [100,101]. In brief, the freeze-dried AuNPR_n_ extracts were digested with nitric and hydrochloric acids (1:3 molar ratio). Yttrium (100 mg/L) was used as an internal standard. According to this procedure, microwave-assisted acid digestion (350 W, 15 s) was combined with ultrasound cycles. Three aliquots of 10 µL of the digested material were analyzed per sample. The elemental concentrations were expressed in mg/mL.

#### 3.3.3. UV-Vis Spectroscopy

The UV-Vis spectra of the AuNPR_n_ samples were recorded in water with a Varian Cary 50 UV/Vis spectrophotometer, at room temperature, in the range 200–900 nm, using either disposable or quartz cuvettes (path length = 1 cm).

#### 3.3.4. Dynamic Light Scattering (DLS) and Zeta-Potential Measurements

The DLS measurements were performed using a Malvern Zetasizer Nano ZS (Malvern Instruments Ltd., Worcestershire, UK) equipped with a 633 nm He-Ne laser and operating at an angle of 173°. The software used to collect and analyze the data was the Dispersion Technology Software (DTS) version 5.10 from Malvern. In total, 600 µL of each sample was measured in low-volume semi-micro disposable sizing cuvettes (Fisher Scientific, USA) with a path length of 10 mm. Triplicate measurements were taken at a position of 4.65 mm from the cuvette wall with an automatic attenuator. For each sample, 15 runs of 10 s were performed. The size distribution, the Z-average diameter and the polydispersity index (PDI) were obtained from the autocorrelation function using the “general purpose mode” for all of the nanoparticle samples. The default filter factor of 50%, the default lower threshold of 0.05 and an upper threshold of 0.01 were used. The zeta potential measurements were performed in triplicates using water as a dispersant and the Huckel model. For each sample, 20 runs were performed in the auto-analysis mode.

#### 3.3.5. Attenuated Total Reflectance Fourier-Transform Infrared Spectroscopy (ATR-FTIR)

The Fourier transform infrared (FTIR) spectra were measured using a FTIR Nicolet iS50 spectrometer equipped with an ATR diamond crystal accessory (Thermo Fisher Scientific, Madison, WI, USA). A drop of each sample was used to cover the ATR crystal, then stirred to disperse the AuNPR_n_ in the aqueous solution. The spectra were recorded at room temperature at a resolution of 4 cm^−1^, with 128 scans from 4000 to 400 cm^−1^.

#### 3.3.6. X-ray Diffraction (XRD)

The crystallinity of the synthesized AuNPR_n_ was checked by powder X-Ray diffraction (PXRD) using a Bruker D2 Phaser diffractometer equipped with a Cu Kα X-ray tube (monochromatic radiation, λ = 1.5406 Å). All of the scans were performed in the 2*θ* range from 10° to 80°, after establishing the following operational settings: a voltage of 30 kV, a current of 10 mA, a step size of 0.02°, and a time per step of 10 s. The scans were collected electronically and processed using the DIFFRAC.EVA pattern processing software. The crystallite size of the AuNPR_n_ samples was estimated by Scherrer’s equation:

$$D=\frac{K\cdot \lambda }{\beta \cdot \cos\theta }$$

where *D* is the estimated value of the crystallite size (nm), λ is the X-ray wavelength (0.154 nm), K is the Scherrer constant of which the value varies from 0.62 to 2.08 (in the absence of any information related to the crystal shape, it is commonly accepted to use K = 0.9), *θ* is the Braggs angle corresponding to the most intense reflection, and β is the full width at the half maximum of the peak (in radians) [84].

#### 3.3.7. Transmission Electron Microscopy (TEM) and Scanning Electron Microscopy (SEM)

The transmission electron microscopy (TEM) images were obtained on a JEOL 1400 transmission electron microscope (JEOL LTD., Tokyo, Japan). The samples for the TEM analyses were prepared by placing 5 µL of the solution containing AuNPs onto a 300-mesh carbon-coated copper grid. The excess amount of the solution was carefully removed, and the carbon-coated copper grid dried for an additional 5 min. TEM image Adobe Photoshop, equipped with Fovea plug-ins, was used to process the obtained images in order to determine the average size and size distribution of the AuNPs. The surface morphology was recorded using a FEG-SEM JEOL 7001 (JEOL LTD., Tokyo, Japan), operating at 15 keV.

#### 3.3.8. DPPH Radical-Scavenging Activity of *N. alba* Extract and AuNPR_n_

According to the DPPH (2,2-Diphenyl-1-picrylhydrazyl) assay, equal volumes of DPPH (50 μM) and the samples (*N. alba* root extract and AuNPR_n_) were mixed in a 96-well microplate and left in darkness at room temperature for 30 min. After that, the absorbance was recorded at 517 nm using the Tecan Pro 200 multiwell plate reader (Tecan, Männedorf, Switzerland). Equal volumes of solvent and DPPH were used to prepare the blank sample. The linear Trolox curve was built using concentrations between 0 and 250 µg/mL, and the results were expressed as equivalents of Trolox (mg TEq/g) per 1 g of the sample. The DPPH inhibition percentage was calculated with the following formula:

$$\mathrm{Inhibitory}\;\mathrm{Percentage}\;\left(\%\right)=\frac{{\mathrm{Blank}}^{\prime }s\;\mathrm{absorbance}-{\mathrm{Sample}}^{\prime }s\;\mathrm{absorbance}}{{\mathrm{Blank}}^{\prime }s\;\mathrm{absorbance}\;}\times 100$$

### 3.4. Antibacterial Activity of Gold Nanoparticles (AuNPR_n_)

The bacterial strains *Staphylococcus aureus* Newman (Gram-positive) and *Escherichia coli* ATCC25922 (Gram-negative), used in this work, were isolated from human infections [102,103]. They were maintained in a Lennox Broth (LB) solid medium composed of tryptone (10 g/L), yeast extract (5 g/L), NaCl (5 g/L), and agar (20 g/L).

The antimicrobial activity of the synthesized AuNPs and the gold salt precursor HAuCl_4_ towards the bacterial strains was assessed by the determination of the Minimal Inhibitory Concentration (MIC) based on previously described standard methods [101,104]. Briefly, stock solutions of the gold nanoparticles or the gold salt precursor were prepared in Mueller–Hinton (MH) broth (Fluka Analytical) at final concentrations of 400 µg/mL. Serial dilutions (1:2) of the stock solutions were prepared for each sample in the MH broth, with final concentrations ranging from 200 to 0.1 μg/mL. Then, 100 μL aliquots of adequately diluted bacterial suspensions of *S. aureus* Newman or *E. coli* ATCC 25922 were mixed with the MH broth serially diluted aliquots of AuNPR_1–5_ or the gold salt, to obtain $5\times 10^{5}$ CFU/mL. The bacterial suspensions were prepared from cultures grown for 5 h in MH broth at 37 °C and 250 rev.min^−1^, and adequately diluted with fresh MH broth. After 22 h of incubation of the bacteria with the AuNPR_1–5_ or the gold salt at 37 °C, the well’s content was resuspended by pipetting, and the OD640 was measured in a SPECTROstar Nano microplate reader (BMG Labtech).

At least three independent experiments were performed in duplicate for each sample under study. The minimum inhibitory concentration (MIC) was defined as the lowest concentration of the antimicrobial agent that inhibited the visible growth of a microorganism after 22 h of incubation. Positive (no sample) and negative controls (no inoculum) were performed for each experiment.

### 3.5. Anticancer Activity of Gold Nanoparticles (AuNPR_n_)

For the evaluation of the anticancer activity of synthesized gold nanoparticles, human ovarian cancer cells A2780 (Sigma-Aldrich, St. Louis, Missouri, USA) were selected. The V79 fibroblasts (ATCC) were used to assess the cytotoxicity in normal cells. The cell culture was prepared using RPMI-1640 (Roswell Park Memorial Institute Medium) (A2780) or DMEM (Dulbecco’s Modified Eagle’s Medium) with Glutamax (V79), supplemented with 10% fetal bovine serum (FBS) and 1% antibiotics. The cells were suspended in a medium, placed in culture flasks and incubated at 37 °C in the presence of CO_2_ (5%). Upon confluence, the cells were removed by treatment with trypsin/EDTA solution, suspended with a complete medium and further diluted for the cell count or sub-culturing. 

An MTT assay was used to evaluate the cellular viability. This assay involves the reduction of the soluble yellow tetrazolium salt to insoluble purple formazan crystals by the succinate dehydrogenase enzyme, which is present in the mitochondria of metabolically active cells. For the assays, the cells were seeded into 96-well plates at a density of 2 × 10^4^ cells/200 µL. After a period of 24 h for adherence, the cells were treated with 200 µL of different concentrations of AuNPR_n_ in the range of 1 to 100 μg Au/mL, and were incubated at 37 °C for 48 h in the presence of CO_2_ (5%). After the incubation, the medium was aspirated and 200 μL MTT solution in PBS (0.5 mg/mL) was added to the cells. The latter were further incubated for another 3 h under the same conditions. Finally, the formazan crystals were dissolved in 200 μL DMSO and the absorbance was measured at 570 nm. The absorbance of the untreated cells was used as the control. The spectrophotometrical absorbance of the purple blue formazan dye was measured with a microplate reader at 570 nm (Power Wave Xs, Bio-Tek, Winooski, VT, USA). The IC_50_ was determined using GraphPad Prism 5 software from dose–response curves.

## 4. Conclusions

For the first time, we showed that the root extracts of *Nymphaea alba* from the Danube Delta Biosphere Reserve enable the stabilization of gold nanoparticles, obtained by a single one-pot reaction using sonochemistry at room temperature, as a simple, economical, efficient and environmentally friendly methodology. The experimental conditions, such as the pH, Au/root extract ratio and reaction time are important parameters to control, as they tune the AuNPR_n_ size and charge. The hydrodynamic size of AuNPR_n_ increases with the Au/root extract ratio: a higher ratio corresponds to a higher hydrodynamic size; on the other hand, the gold core (average crystallite size) characterized by PXRD did not show significant differences, and presented a crystal size inferior to 20 nm.

All of the nanoparticles have high negative zeta potential, from −62 ± 11 mV to −46 ± 7 mV. The AuNPR_n_ presented some shape heterogeneity and moderate monodisperse distribution, as shown by the TEM and SEM analysis. The ATR-FTIR indicated that AuNPR_n_ interact mainly with hydroxyl groups present in the polyphenol compounds, in addition to the flavonoids, tannins and other molecules present in the extracts and participating in the reduction of Au^3+^ to Au^0^ and its stabilization. The analysis of the FTIR spectra also confirmed the very weak concentration of the flavonoids and polyphenols in the AuNPR_2_ sample, in agreement with the spectrophotometric analysis results. As previously observed for other nanoparticles biosynthesized with plant extracts, the SEM images showed that the nanoparticles exhibited a surrounding matrix formed of extracted material that exerts a protective and stabilizing function, preventing their aggregation.

Apart from AuNPR_2_, all of the nanoparticles showed a high antioxidant capacity, superior to that observed for the root extract. The stabilized gold nanoparticles exhibited size-dependent antibacterial and anticancer activity, both increasing with decreasing particle sizes. In fact, the largest AuNPs (AuNPR_2_ and AuNPR_5_) were the less biologically active nanoconstructs. Besides the size, this reduced activity certainly also reflects their low content of polyphenols.

To sum up, this work focused on gold nanoparticles from root extracts of Danube Delta *Nymphaea alba* with dual antimicrobial and anticancer activity. This multifaceted performance, moderate selectivity and reduced toxicity can position them as effective therapeutic agents, with the added benefit of a low environmental impact.

In future works, these AuNPR_n_, in particular AuNPR_3_ and AuNPR_4_, will also be exploited as potential candidates for applications as drug carriers owing to their biological properties, i.e., their anticancer activity against A2780 cisplatin-sensitive ovarian cancer cells, while retaining some antibacterial activity. The AuNPR_3/4_ loaded with anticancer drugs or herbal medicines for the active targeting of ovarian cancer could improve their biological profiles in vitro and in vivo, acting through different mechanisms that can reduce the development of cancer drug resistance, reduce systemic drug toxicity and improve the therapeutic outcomes.

## Figures and Tables

**Table 1:** Reaction conditions used in the synthesis of AuNPR_n_ (n = 1–5).

AuNPR_n_	HAuCl_4_ (mM)	Root Extract (mg/mL)	Reaction Volume (mL)	pH	Sonication Time (min)
AuNPR_1_	1.5	5.47	15.24	7.0	10
AuNPR_2_	1.5	7.39	20.30	6.4	10
AuNPR_3_	1.5	7.38	20.32	8.4	40
AuNPR_4_	2.0	8.16	18.38	7.8	40
AuNPR_5_	3.0	5.54	15.04	7.8	40

**Table 2:** Total polyphenols, flavonoids and condensed tannins from *N. alba* root extract and AuNPR_n_. Abbreviations: GA, gallic acid; TA, tannic acid; Q, quercetin; R, rutin; C, catechin; Eq, equivalent.

Sample	Total Polyphenols		Total Flavonoids		Total Condensed Tannins
	(mg GAEq/g Sample)	(mg TAEq/g Sample)	(mg QEq/g Sample)	(mg REq/g Sample)	(mg CEq/g Sample)
R extract	572.16 ± 4.91	606.35 ± 5.26	22.35 ± 0.96	14.38 ± 0.97	1.70 ± 0.13
AuNPR_1_	39.65 ± 1.43	42.08 ± 1.50	15.52 ± 0.82	10.03 ± 0.89	0.04 ± 0.00
AuNPR_2_	0.43 ± 0.05	0.46 ± 0.03	-	-	-
AuNPR_3_	28.89 ± 0.99	30.55 ± 1.23	5.22 ± 0.33	3.17 ± 0.26	0.02 ± 0.00
AuNPR_4_	33.00 ± 1.17	34.95 ± 2.01	-	-	-
AuNPR_5_	22.84 ± 0.86	24.07 ± 0.94	7.78 ± 1.45	4.87 ± 0.72	0.03 ± 0.00

- Negative/unidentified. R extract—*N. alba* root extract.

**Table 3:** Gold content, root extract content, hydrodynamic size, zeta potential and Au/root extract ratios of the AuNPR_n_.

Sample	[Au] * mg/mL	Ratio Au/Root Extract	SPR λ_max_ (nm)	Hydrodynamic Size (PDI) (nm)	Zeta Potential (ζ) (mV)
AuNPR1	3.05 ±0.15	0.56	625	280.2 (0.23)	−52 ± −7
AuNPR2	3.23 ±0.65	0.44	587	150.0 (0.2)	−46 ± −7
AuNPR3	2.81 ± 0.14	0.38	618	60.7 (0.22)	−62 ± −11
AuNPR4	1.94 ± 0.10	0.24	601	32.3 (0.35)	−56 ± −9
AuNPR5	4.04 ± 0.20	0.73	628	209.8 (0.28)	−60 ± −9

* Determined by PIXE.

**Table 4:** PXRD and TEM characterization of the AuNPR_n_ samples.

Sample	XRD/Unit Cell		Crystallite/Particle Size (nm)	
	Phase	Lattice Parameter (Å) *	XRD	TEM
AuNPR_1_	Metallic Au	4.0756	19.6 ± 0.9	62.2 ± 13.0
AuNPR_2_	Metallic Au	4.0782	16.3 ± 0.6	63.2 ± 8.2
AuNPR_3_	Metallic Au	4.0734	15.2 ± 0.7	49.3 ± 7.2
AuNPR_4_	Metallic Au	4.0891	16.1 ± 0.6	38.2 ± 4.4
AuNPR_5_	Metallic Au	4.0757	18.5 ± 0.9	68.0 ± 10.1

* Lattice parameter of the Crystallography Open Database (COD 9008463), a = 4.0782 Å.

**Table 5:** Antioxidant activity (inhibition percent) of the *N. alba* root extract and AuNPR_n_ samples determined by the DPPH method.

Sample	Inhibition Percent (%)
R extract	72.20 ± 0.33
AuNPR_1_	95.77 ± 1.25
AuNPR_2_	56.47 ± 2.03
AuNPR_3_	92.38 ± 2.54
AuNPR_4_	94.29 ± 3.14
AuNPR_5_	90.05 ± 0.99

R extract—*N. alba* root extract.

**Table 6:** Estimated MIC values of the AuNPR_1–5_ and HAuCl_4_ precursor towards *S. aureus* Newman and *E. coli* ATCC25922. The results are the mean of three independent experiments performed with two replicates.

	MIC (µg Au/mL)					
	AuNPR_1_	AuNPR_2_	AuNPR_3_	AuNPR_4_	AuNPR_5_	HAuCl_4_
*S. aureus* Newman	200	>200	200	100	>200	50
*E. coli* ATCC25922	>200	>200	200	200	>200	6.25

## Supplementary Materials

The following are available online at https://www.mdpi.com/article/10.3390/nano11061562/s1. Figure S1: HPLC-DAD chromatogram of *N. alba* root extract with detection at 300 nm; Table S1: HPLC-DAD identification and quantification of polyphenols from the *N. alba* root extract; Figure S2: Size distribution plot of AuNPR_n_ (n =1 –5) by DLS analysis; Table S2: Hydrodynamic sizes of the AuNPR_n_; Figure S3: XRD patterns of the AuNPR_n_ samples; Figure S4: TEM images of AuNPR_2_ and AuNPR_3_; Figure S5: Dose–response curves obtained using the GraphPad Prism software to determine the IC_50_ values for AuNPR_n_ upon incubation with the A2780 cells (a) and the V79 cells (b) for 48 h.
