# Supplementary material for: Sono-Biosynthesis and Characterization of AuNPs from Danube Delta Nymphaea alba Root Extracts and Their Biological Properties
Source: Nanomaterials (Basel). 2021 Jun 14;11(6):1562. doi: 10.3390/nano11061562 (PMC8231883; doi:10.3390/nano11061562)
Supplement: Supplementary file 1 [file nanomaterials-11-01562-s001.zip › nanomaterials-1238913-supplementary.pdf]

Sono-biosynthesis and characterization of AuNPs from Danube Delta *Nymphaea alba* root extracts and their biological properties

Mihaela Cudalbeanu [1,2], David Peitinho [3], Francisco Silva [3], Rosa Marques[3,4], Teresa Pinheiro [4,5], Ana C. Ferreira [6], Fernanda Marques [3,4], António Paulo [3,4], Catarina F. Soeiro [5], Sílvia Andreia Sousa [5], Jorge Humberto Leitão [5], Aurel Tăbăcaru [1], Sorin Marius Avramescu [2,7], Rodica Mihaela Dinica [1,*] and Maria Paula Cabral Campello [3,4,*]

[1] Faculty of Sciences and Environment, Department of Chemistry Physical and Environment, "Dunărea de Jos" University of Galati, 111 Domnească Street, 800201 Galati, Romania; mihaela.cudalbeanu@ugal.ro (M.C.); aurel.tabacaru@ugal.ro (A.T.); Rodica.Dinica@ugal.ro

[2] Research Center for Environmental Protection and Waste Management, University of Bucharest, 91–95 Splaiul Independentei, 050095 Bucharest, Romania; mihaela.cudalbeanu@ugal.ro (M.C); sorin_avramescu@yahoo.com (S.M.A.)

[3] Centro de Ciências e Tecnologias Nucleares, Instituto Superior Técnico, Universidade de Lisboa, Campus Tecnológico e Nuclear, Estrada Nacional 10, Km 139.7, 2695-066 Bobadela LRS, Portugal; dpeitinho@gmail.com (D.P.); fsilva@ctn.tecnico.ulisboa.pt (F.S.); rmarques@ctn.tecnico.ulisboa.pt (R.M.); fmarujo@ctn.tecnico.ulisboa.pt (F.M.); apaulo@ctn.tecnico.ulisboa.pt (A.P.); pcampelo@ctn.tecnico.ulisboa.pt (M.P.C.C.)

[4] Departamento de Engenharia e Ciências Nucleares (DECN), Instituto Superior Técnico, Universidade de Lisboa, Estrada Nacional 10, 2695-066 Bobadela LRS, Portugal; rmarques@ctn.tecnico.ulisboa.pt (R.M.); teresa.pinheiro@tecnico.ulisboa.pt (T.P.); fmarujo@ctn.tecnico.ulisboa.pt (F.M.); apaulo@ctn.tecnico.ulisboa.pt (A.P.); pcampelo@ctn.tecnico.ulisboa.pt (M.P.C.C.)

[5] iBB-Institute of Bioengineering and Biosciences, Department of Bioengineering, Instituto Superior Técnico, University of Lisbon, 1049-001 Lisbon, Portugal; teresa.pinheiro@tecnico.ulisboa.pt (T.P.); catarina.soeiro@tecnico.ulisboa.pt (C.F.S.); sousasilvia@tecnico.ulisboa.pt (S.A.S.); jorgeleitao@tecnico.ulisboa.pt (J.H.L.)

[6] Centro de Química Estrutural, Instituto Superior Técnico, Universidade de Lisboa, Campus Tecnológico e Nuclear, Estrada Nacional, Estrada Nacional 10, Km 139.7, 2695-066 Bobadela LRS, Portugal; acferreira@ctn.tecnico.ulisboa.pt

[7] Faculty of Chemistry, Department of Organic Chemistry, Biochemistry and Catalysis, University of Bucharest, 90–92 Soseaua Panduri, 050663 Bucharest, Romania;

* Correspondence: rodica.dinica@ugal.ro (R.M.D.); pcampelo@ctn.tecnico.ulisboa.pt (M.P.C.C.)

The HPLC-DAD chromatographic separation and identification technique of total polyphenol compounds based on the retention time of standard references showed in the *N. alba* ethanolic root extract the presence of gallic acid, epicatechin (-), p-coumaric acid, daidzein, rutin, hyperoside and naringin (Figure S1). Our previous studies showed the presence of 20 polyphenol compounds in the *N. alba* methanolic root extract such as HHDP-hexoside, quinic acid, corilagin, vanillic acid, castalin, gallic acid, caffeic acid, p-coumaric acid, tannic acid, rutin, ellagic acid, ellagic rhamnosyl acid, naringenin, naringin, ferulic acid, catechin, epicatechin, apigenin, brevifolin and orientin [47].

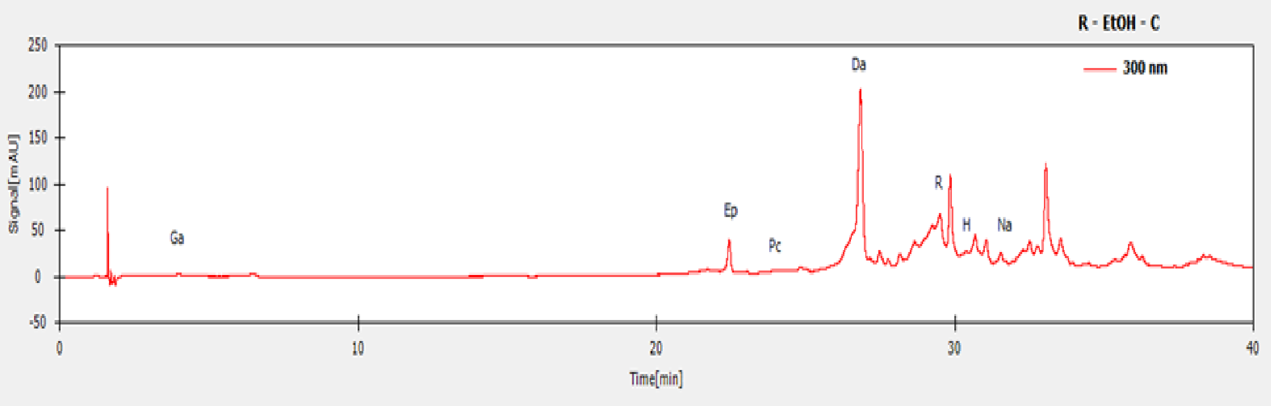

Figure S1. HPLC-DAD chromatogram of *N. alba* root extract with detection at 300 nm. Peaks identified were Ga – gallic acid, Ep – epicatechin, Pc - p-coumaric acid, Da – daidzein, R – rutin, H – hyperoside, Na – naringin.

The quantification of polyphenol compounds from the *N. alba* root extract are illustrated in the Table S1. Of these it distinguish epicatechin (-) which has the highest concentration (119.11±3.04 mg/kg), followed by daidzein (28.96±0.99 mg/kg) and rutin (9.11±0.28 mg/kg).

Table S1. HPLC-DAD identification and quantification of polyphenols from the *N. alba* root extract.

**Peak**	**Compound**	**T_R* (min)**	**T_R** (min)**	**Amount (mg/kg)**
**Ga**	Gallic acid	3.78	3.99	2.05±0.01
**Ep**	Epicatechin (-)	23.02	23.15	119.11±3.04
**Pc**	p-Coumaric acid	24.06	24.09	2.50±0.01
**Da**	Daidzein	26.44	26.65	28.96±0.99
**R**	Rutin	29.69	29.72	9.11±0.28
**H**	Hyperoside	30.60	30.79	0.98±0.01
**Na**	Naringin	31.55	31.56	2.01±0.01

* Retention time (T_R) mean error for standard references was ± 0.0001–0.2 min.
** Retention time (T_R) mean error for compounds was ± 0.0001–0.2 min.

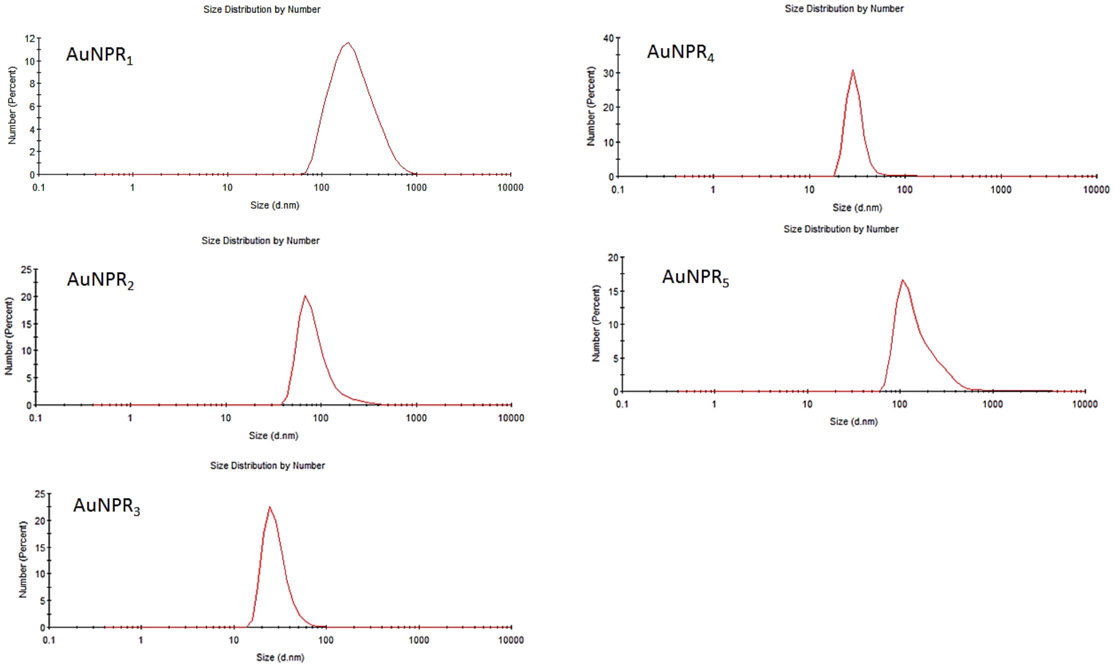

Figure S2. Size distribution plot of AuNPRn (n=1-5) by DLS analysis.

Table S2. Hydrodynamic sizes of the AuNPRn.

	**Hydrodynamic size (PDI) (nm)**
AuNPR1	280.2 ± 27.6 (0.23 ± 0,01)
AuNPR2	150.0 ± 29.8(0.20±0,04)
AuNPR3	60.7± 5.4(0.22 ± 0,01)
AuNPR4	32.3 ± 6.7(0.35 ± 0,01)
AuNPR5	209.8± 41.6(0.28 ± 0,02)

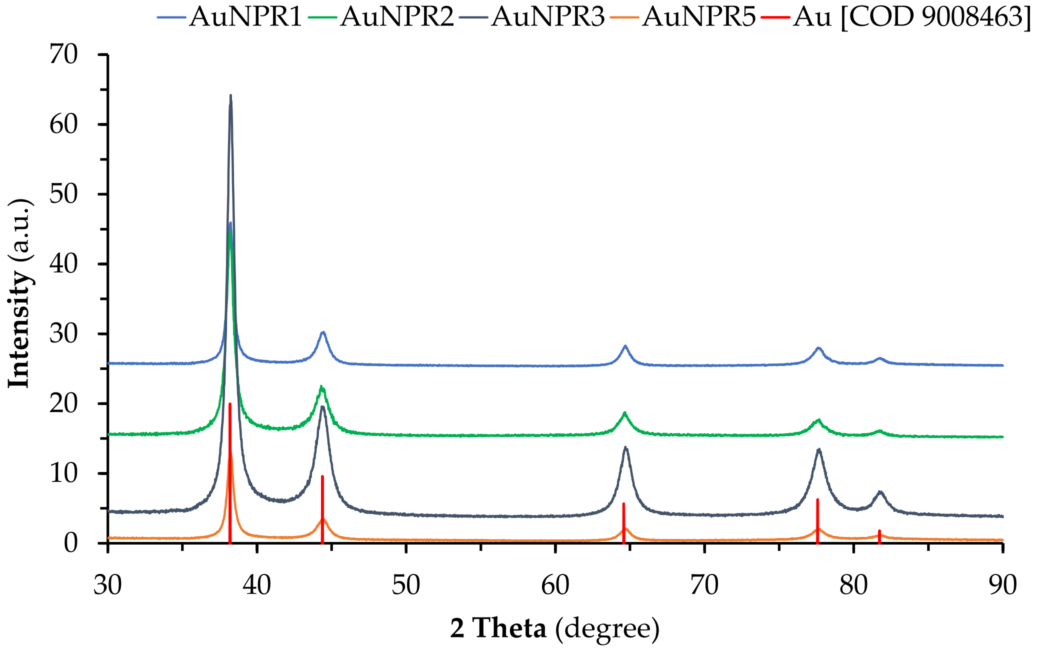

Figure S3. XRD patterns of AuNPRn samples.

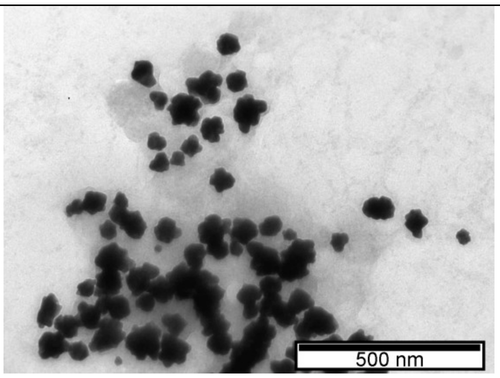

(a)

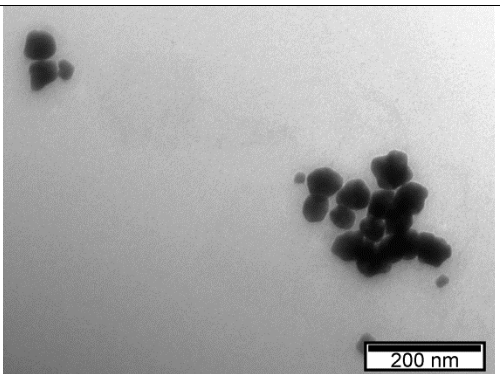

(b)

Figure S4. TEM images of $AuNPR_2$ (a), $AuNPR_3$ (b).

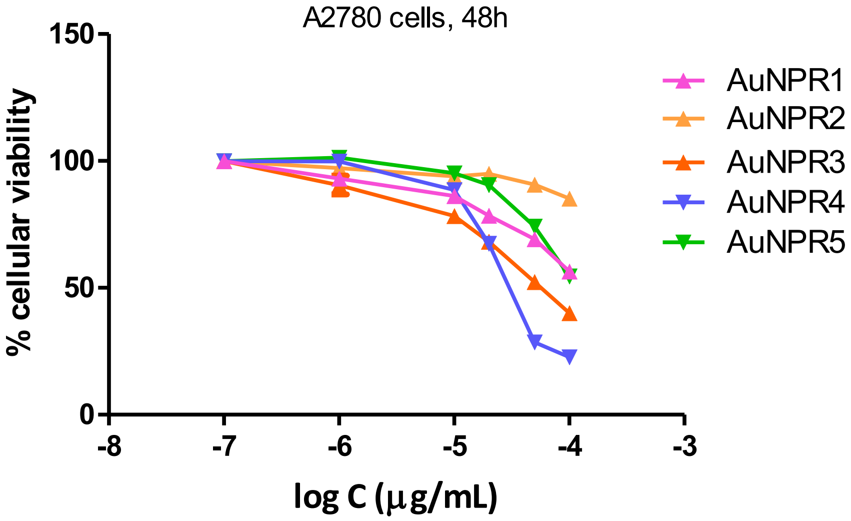

a)

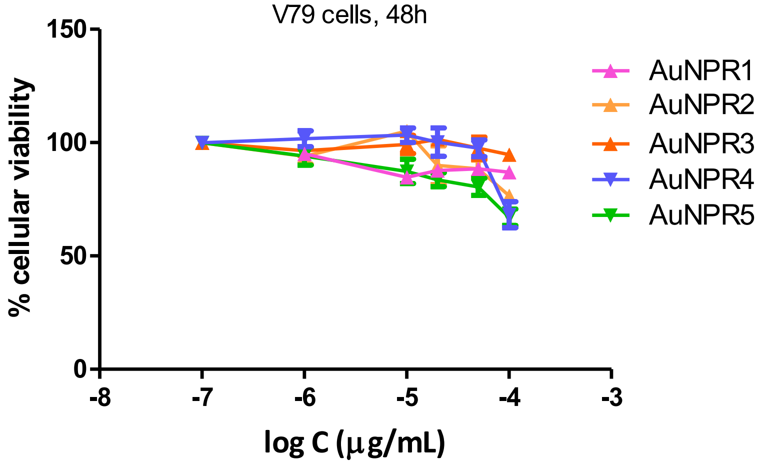

b)

Figure S5. Dose-response curves obtained using the GraphPad Prism software to determine the IC_{50} values for $AuNPR_n$ upon incubation with the A2780 cells (a) and the V79 cells (b)for 48h.
